# Advanced Stage Breast Sarcoma Treatment in a Third World Country Public Hospital

**DOI:** 10.1155/2018/4985012

**Published:** 2018-08-06

**Authors:** Amanda Lino de Faria, Cinthia Moreno Garcia, Gabriela de Andrade Rodrigues, Lais Helena Dumbra Toloni dos Santos, Gabriela B. K. Uyeda, Simone Elias, Marair G. F. Sartori, Afonso Celso Pinto Nazário, Gil Facina

**Affiliations:** ^1^Federal University of São Paulo (UNIFESP), São Paulo, Brazil; ^2^Department of Gynecology, Federal University of São Paulo (UNIFESP), São Paulo, Brazil

## Abstract

The case reports a 49-year-old patient, drug-addicted, undernourished, and homeless, who was referred to our service presenting a diagnostic of breast sarcoma and ulcerating tumor which extended from the right breast to the right flank. She underwent hygienic mastectomy and, as it developed, she presented a range of complications, culminating in the recurrence of the tumor and pulmonary metastasis few months after her initial treatment. There is relevance in our study not only because it reports the development of the breast sarcoma, rare neoplasm, and its aggressiveness with fast recurrence, but also because it exposes the impact of biopsychosocial behavior of this patient in her clinical outcome.

## 1. Introduction

Nowadays, breast cancer is the most common malignant cancer in Brazilian women, excluding nonmelanoma skin cancer, while soft tissue sarcomas are rare tumors derived from mesenchymal cells, comprising 1% of the malignant neoplasms in the adult population [1].

The highest incidence of soft tissue sarcomas is after the fifth decade of the life. Tumors appear as a firm and high volume painless mass, with a rapid growth that reaches around 5 to 6 cm. In circumstances in which there is a tardy diagnosis, these tumors can outgrow to the skin and ulcerate, causing bleeding and infection. The best prognostic predictor is the tumor size since masses smaller than 5 cm have a better prognostic [2].

Considering it a rare neoplasm, the diagnosis of sarcoma is not always easy and it is made by histopathology after removing the lesion [3]. The treatment is guided by the histological grade and adequacy of the surgical margins. Surgery with clear surgical margins is the initial treatment recommended for most patients, and the adjuvant chemotherapy and radiotherapy are individualized according to each patient.

## 2. Case Report

**Figure 1:**
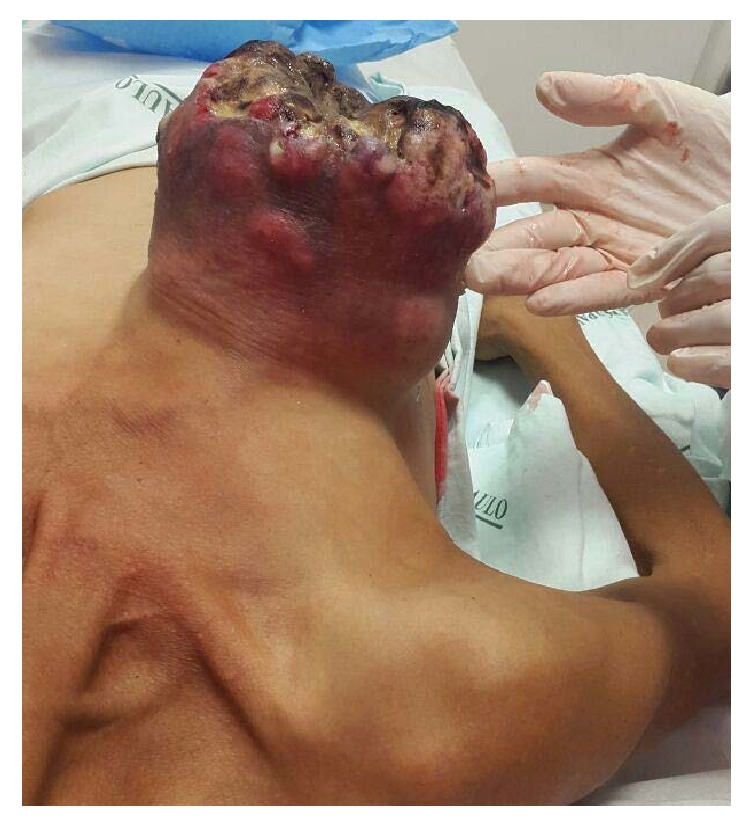
Aspect of the lesion in the admission of the patient.

**Figure 2:**
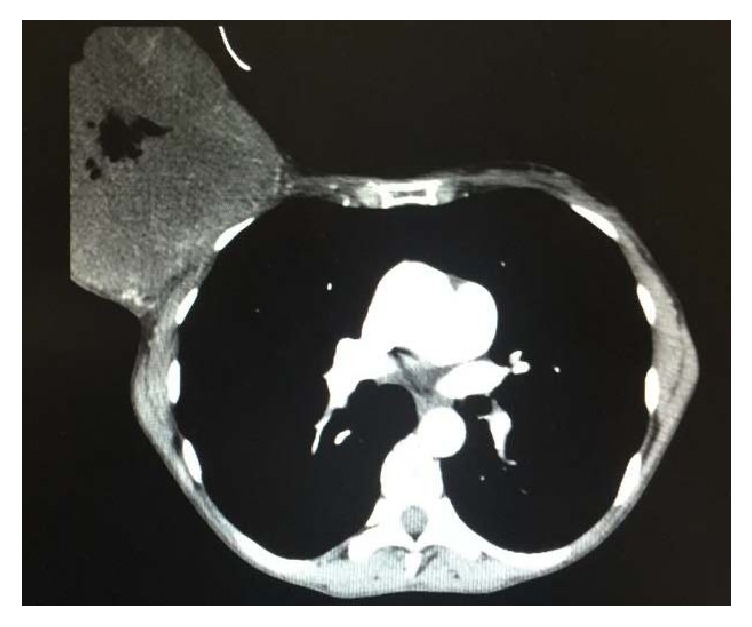
Chest tomography at the admission.

The case reports patient CAGF, female, 49 years old, homeless in São Paulo, crack addict for ten years, and smoker 70 years/pack of cigarettes, G10P10, without breast cancer in her family history. She mentioned that three years ago she noticed a progressive increase of her right breast and the appearing of bleeding ulcers. She noted a not measured ponderal loss and progressive weakness. She sought primary healthcare service for the first time three months before where a biopsy of the lesion was performed. The anatomopathological examination evidenced an atypical fusiform proliferation, ulcerated and necrotic. The patient was referred to the São Paulo Hospital with a bulk tumor mass, which extended from the right breast to the right flank, friable, bleeding, and sore with a malodorous (Figure 1). She was undernourished (BMI 15,57/m^2^), in a regular, state feverish and pale +/4+. Her physical examination performed by medical equipment did not show alterations. The chest tomography showed the cystic injury and lungs without signs of metastasis (Figure 2).

**Figure 3:**
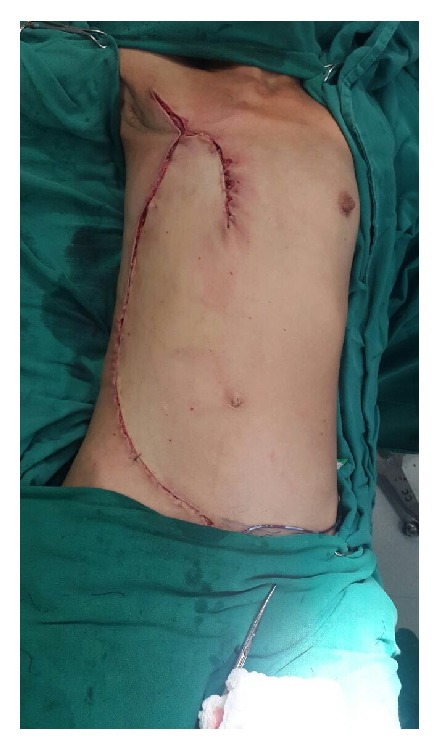
Immediate postoperative.

Initially, due to the infectious character of the wound, antibiotic therapy was performed with intravenous clindamycin. After a discussion of the medical board, a hygienic mastectomy, and reconstruction unilateral thoracoabdominal, the surgical specimen performed had the following dimensions: 14,5x12x9 cm and 1.375g (Figure 3).

The anatomopathological exam resulted in a malignant mesenchymal tumor of a high histological grade. The immunohistochemistry showed pleomorphic undifferentiated sarcoma of high grade (Ki-67 positive in 70% of the sample, negative CD34, negative S-100, and negative vimentin).

**Figure 4:**
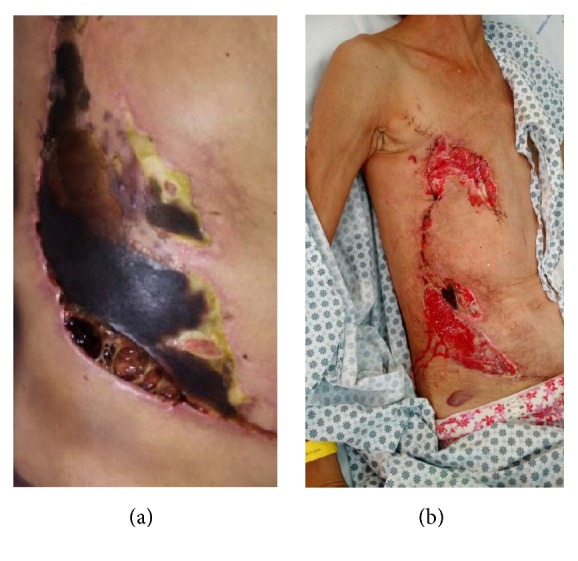
Necrosis of the surgical flap (a) and final aspect after debridement (b).

Two weeks after the surgical procedure (14° PO), the patient evolved with necrosis in part of the thoracoabdominal flap; it was necessary to perform the debridement of the necrotic area (Figures 4(a) and 4(b)). On the 26° PO, a new debridement of the surgical wound was performed applying skin graft from the right thigh.

During the hospital stay, the patient presented symptoms and laboratory aspects of anemia, being necessary transfusion with red blood cells. The antibiotic therapy with ceftriaxone and metronidazole was staged for clindamycin (POI) and cephalothin (12° PO) and, later, for piperacillin and tazobactam (17th PO) due to remaining infectious signs in the surgical wound.

After 21 days of antibiotic therapy with piperacillin and tazobactam (39° PO), the patient developed fever (40,3°C), tachycardia, and sweating progressing to a decreased level of consciousness. The patient was transferred to the ICU, where she remained for 48 hours, due to a sepsis of unknown origin and neutropenia of 146 U/L, secondary to sepsis. She initiated meropenem and vancomycin remaining stable, without the use of vasoactive drugs, but maintaining the fever. Infectious screening was performed without the identification of origin.

After 48 hours of admission in the UCI, the patient had an improvement in its fever and neutropenia status, being transferred back to the Gynecology Ward where she presented diffuse maculopapular rash over all integument and face worsening after the contrasted CT on the following day. Vancomycin was suspended due to a suspicion of the pharmacodermy, which was confirmed in skin biopsy.

The patient evolved with a rapid and significant recovery of her skin rash without the need of continued corticotherapy after suspending the vancomycin. Then, the linezolid was initiated to cover the Gram + germs.

After 14 days of meropenem and 7 days of linezolid, the patient presented a satisfactory progression, remaining afebrile and asymptomatic for 12 days.

**Figure 5:**
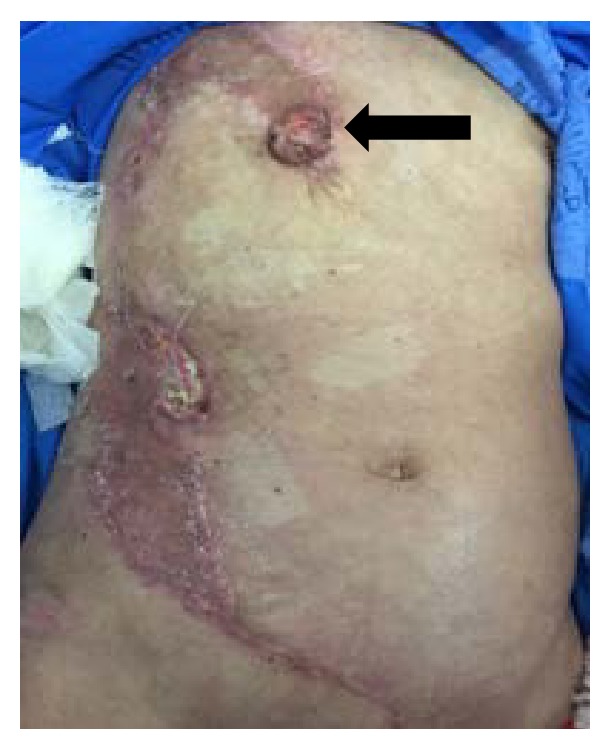
Local recurrence (arrow).

**Figure 6:**
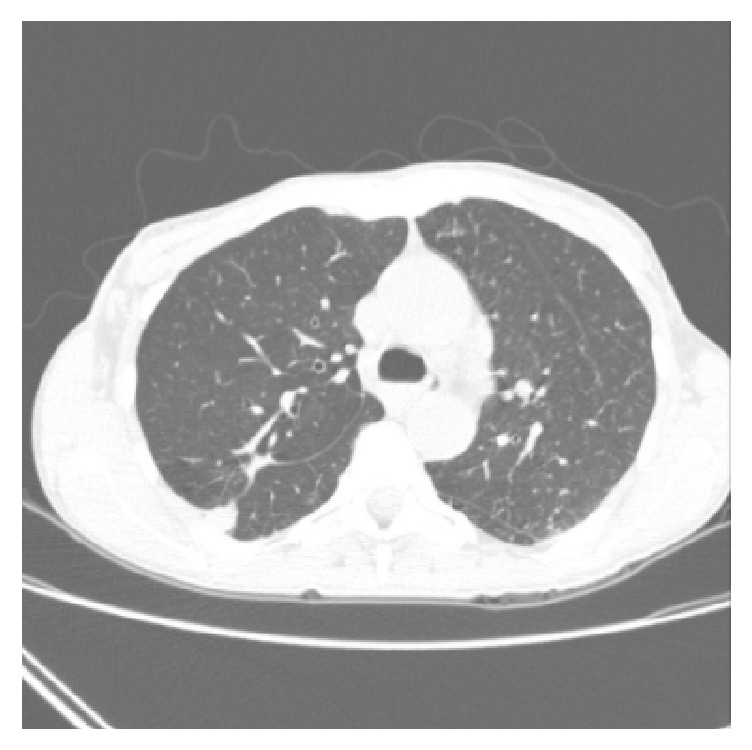
Chest tomography at the hospital discharge.

Despite the clinical improvement and stability, on the 38° PO clinical staff noted the appearance of ulcerated nodule of around 1 cm of diameter in in the right parasternal region, suggestive of local recurrence, which increased progressively, presenting a measure of around 3 cm at the moment of the patient discharge (Figure 5). Besides, during the hospitalization, small pulmonary lesions were identified on computed tomography, absent at the moment of the diagnosis, suggestive of tumor metastasis (Figure 6).

The case was followed and discussed with the Clinical Oncology, which opted to perform outpatient chemotherapy, with Doxorubicin, due to the impossibility of another surgical intervention at the moment. Resources such as transport and psychological follow-up with CAPS (Psychosocial Attention Center) were provided for the adherence and maintenance of the treatment, besides management by the infectious and plastic surgery staff.

**Figure 7:**
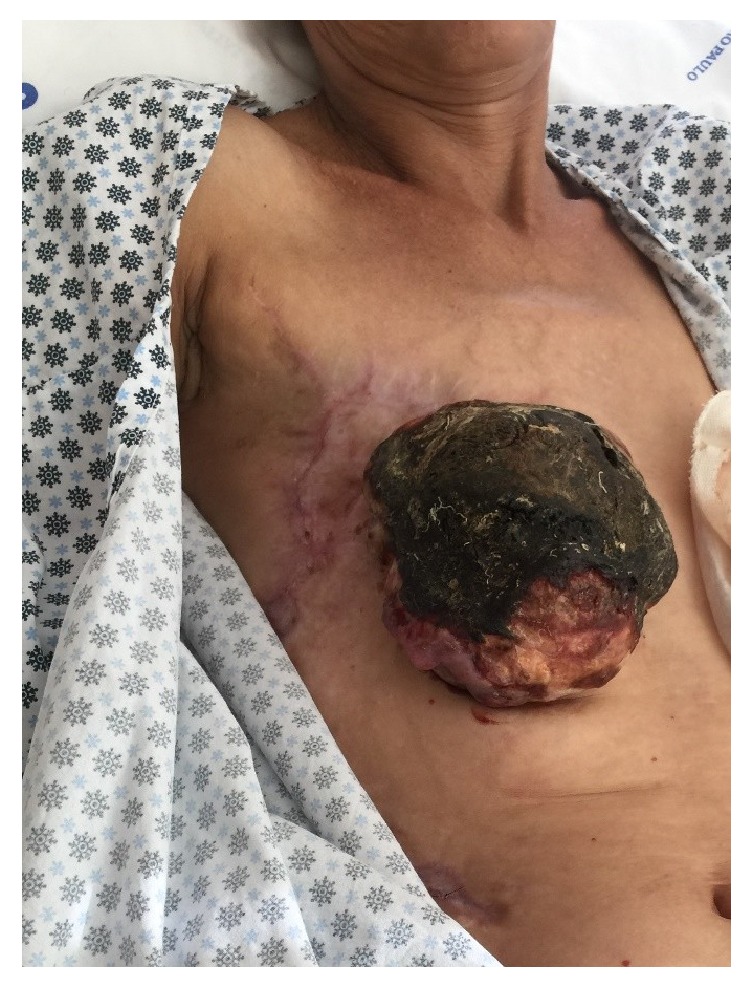
An aspect of the lesion in the readmission of the patient.

During the outpatient follow-up, 4 chemotherapy cycles were performed, but the recurrence progression was maintained, returning 4 months after the hospital discharge, with an extensive lesion, fever, and local refractory pain, with diagnostic hypothesis of sepsis of cutaneous origin (Figure 7).

**Figure 8:**
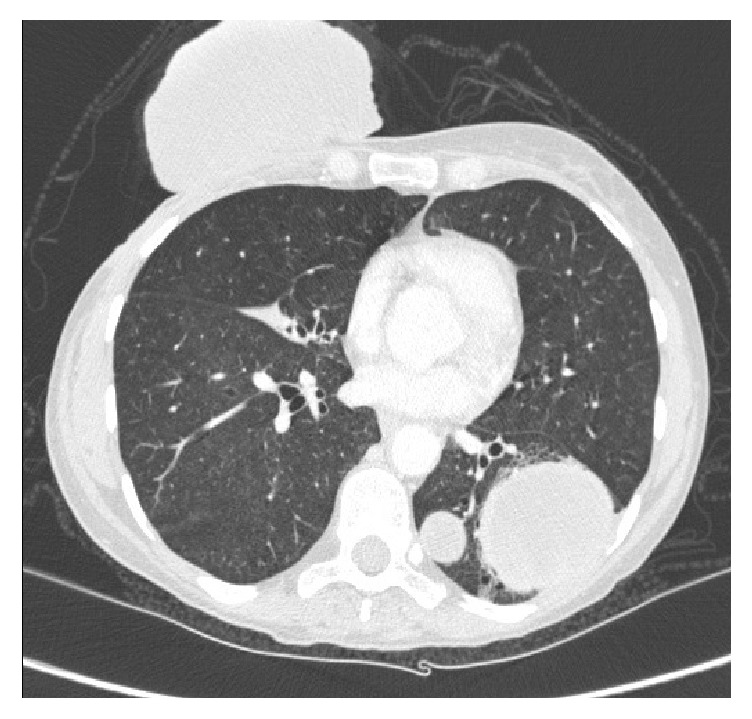
Chest tomography in the rehospitalization.

The antibiotic therapy was initiated, and the patient was hospitalized for stabilization. A new tomography was performed, presenting pulmonary extensive metastatic lesions, bilateral, besides the massive lesion (Figure 8). The patient remained hospitalized for six days, without complications, and introduced to the palliative care after the hospital discharge.

## 3. Discussion

Breast sarcoma, as the primary site, is extremely rare, being found in very few cases like this one of our patient. Along with being rare, it is an aggressive tumor, considering that the overall survival in five years ranges between 50% and 66% and the disease-free survival between 33% and 52%. The best prognostic predictor is the tumoral size, considering lesions smaller than 5 cm as the ones with a more favorable prognostic. The bigger the lesions, the worse the disease prognostic that metastasizes hematogenously [4]. Therefore, it is crucial that its detection happens in the early stages, allowing the initial institution of the treatment, with high chances of cure [5]. As most of the disease recurrence is local, the surgery is the recommended first treatment, whether conservative, respecting the margins, or radical.

In the case of our patient, the social condition and the difficulty to access some health care end up in a late diagnosis which shows extensive and infected lesions. Concomitant to the unfavorable external factors, the anatomopathological and immunohistochemical characteristics confirm the aggressiveness of the tumor and the reserved prognostic. Due to the size and the condition of the lesion, the therapeutic surgery could not be performed; then it is recommended to perform a hygienic mastectomy to reduce the morbidity of the lesion and enable a posttreatment.

The evolution on the postsurgical, with necrosis of the flap and neutropenia, precludes the use of adjuvant therapy. The use of chemotherapy is controversial, having few comparative studies to prove its efficiency in increasing the survival expectation [2, 6], while the radiotherapy may be performed in the sarcoma of high grade, being associated with the decrease of the probability of recurrence and dissemination, being the lung the most affected place [5, 7, 8]. However, although the adjuvant therapy is of extreme importance, the clinic conditions of the patient limited the use of those treatments as well as its prognostic and made recurrence progression easier.

In conclusion, we had an uncommon experience, mentioning not only the rare neoplasm but also facing a differentiated clinic course ahead of limitations that are inherent to the social and clinic condition of the patient. Both culminated in a series of nonideal events, creating clinical evolution permeated by complications which took to an unfavorable outcome, showing quick recidivism of the tumor and few therapeutic possibilities. However, even in this situation, it was possible to make a wide approach, with improvement of the clinical condition of the patient, enabling the outpatient follow-up in the attempt of evaluating the best therapeutic possibilities. Nevertheless, considering the complications, recurrence and metastatic lesions, today the patient presents a reserved prognosis being in palliative care.
